# A Review of High Dose Antipsychotic Therapy Prescribing in a General Adult Community Mental Health Team in Liverpool

**DOI:** 10.1192/bjo.2026.11740

**Published:** 2026-06-30

**Authors:** Lisa Bryant, Phillipa Ward, Stephen Blenkin

**Affiliations:** Mersey Care NHS Foundation Trust, Liverpool, United Kingdom

## Abstract

**Aims:**

High dose antipsychotic therapy (HDAT) refers to prescribing a single antipsychotic at a dose above the maximum British National Formulary (BNF) limit, or prescribing more than one antipsychotic in combination where the combined percentage of each drug’s maximum BNF dose adds up to more than 100%. The potential additional benefit of surpassing recommended doses of antipsychotic therapy is limited and may be associated with higher rate of adverse effects. HDAT should only be used as a last resort after all other treatments have been exhausted, and the benefits must outweigh the risks. The decision to initiate and continue HDAT prescribing must be clinically justified and robustly documented on a regular basis. The audit aimed to assess compliance of a local community mental health team (CMHT) against trust procedure which requires regular clinical review and physical health assessments to be completed every 6 months.

**Methods:**

A retrospective audit was completed of 40 patients who were prescribed HDAT on a single date in February 2025 when the caseload was accessed. Data was collected on clinical reviews, physical investigations and risk assessment forms, using the trusts electronic patient record and shared electronic GP records. Data was collected using a standardised audit tool and analysed in Microsoft Excel.

**Results:**

Of 40 patients prescribed HDAT, 26 (65%) had a review of HDAT completed within the past 6 months. 8 out of 26 reviews (31%) included justification for the continued use of HDAT. Risk assessment forms were completed for 1 out of 26 (4%) patients who had been reviewed. 339 out of 519 (65%) of all physical health assessments were completed in the last 6 months. Overall compliance with trust procedure was 60%.

**Conclusion:**

Adherence to trust standards for prescribing of HDAT was below target. Possible factors identified included difficulty identifying and tracking patients on HDAT, and a lack of system in place for carrying out routine physical health assessments. Recommendations include the creation and maintenance of a live HDAT register, collaboration with physical health team and increasing the awareness of standardised risk assessment forms amongst clinicians.

